# High Prevalence of *Toxoplasma gondii* Infection in Type I Diabetic Patients

**DOI:** 10.1155/2021/8881908

**Published:** 2021-02-09

**Authors:** Qasem Asgari, Mohammad Hossein Motazedian, Amir Khazanchin, Davood Mehrabani, Shahrbanou Naderi Shahabadi

**Affiliations:** ^1^Department of Parasitology and Mycology, Shiraz University of Medical Sciences, Shiraz, Iran; ^2^Stem Cell and Transgenic Technology Research Center, Shiraz University of Medical Sciences, Shiraz, Iran

## Abstract

**Background:**

Type I diabetes (T*Ι*DM) is a genetic or autoimmune disorder, which may be stimulated by induced immune system components due to the underlying infectious diseases. This study was undertaken to find out any possible association between *Toxoplasma gondii* infection and TIDM.

**Materials and Methods:**

One hundred and eighty-two blood samples were taken from individuals who were referred to outpatient clinics in Shiraz city, Southern Iran, during a 6-month period. The age of type I diabetic subjects (*n* = 91) and the control group (*n* = 91) was identical, which were less than 30 years. The sera were examined for IgG and IgM antibodies by ELISA and correlated with epidemiological factors such as age, sex, and family history of diabetes.

**Results:**

Out of 91 diabetic patients, 54 (59.3%) were female and 37 (40.7%) were male. The highest frequency of diabetes belonged to 6-10- and 11-15-year groups (*P* = 0.17). *Toxoplasma* infection prevalence in diabetic and control groups was 28.6% and 7.7%, respectively (*P* = 0.001). A significantly positive family history of diabetes was observed between diabetic patients (31 cases, 34.1%) and the control group (3 cases, 3.3%) (*P* = 0.01). Interestingly, IgG positivity was seen in 13 cases (41.9%) of patients with positive family history of type I diabetes and 13 cases (21.7%) of subjects with no positive family history of type I diabetes (*P* = 0.04).

**Conclusion:**

Our study showed a higher prevalence of *Toxoplasma* infection in type I diabetes patients. It is likely that the prevalence of TIDM decreases by increasing hygiene and preventing toxoplasmosis.

## 1. Introduction

Type I diabetes mellitus (TIDM), previously known as juvenile diabetes, can actually happen at any age. In this autoimmune disease, very little or no insulin is produced by the pancreas. This autoimmune disorder which has a genetic background may be triggered by an immune stimulus. Infiltration of autoreactive CD4+ and CD8+ T-cells due to the immune response against insulin-producing *β-*cells is considered as the main factor in the progression of type I diabetes [1, 2].

Infectious agents such as viruses that target pancreatic cells can lead to infiltration of inflammatory cells and may correspond to the initial steps in the stimulation of autoimmunity. In viral infections, unmasking of *β-*cells may occur resulting in activation of CD8+ T-cells due to recognition and promotion in the production of interferon and upregulation of class I major histocompatibility complex (MHC) molecules of *β-*cells [3].

Recently, research on different mouse models showed initiation of diabetes caused by viral glycoprotein or nucleoprotein from lymphocytic choriomeningitis virus. It required a critical mass of autoreactive T-cells along with activated APCs, and it is possible that, in humans, such a mass is provided progressively over life by repeated or sustained viral infections [4, 5].

Interestingly, *Toxoplasma gondii*, an intracellular parasite infection, induces inflammatory cells such as CD4+ and CD8+ T-cells, monocytes, macrophages, neutrophils, and NK and dendritic cells to produce cytokines such as IL-12, INF-*γ*, TNF-*α*, and NO which participate in the inflammatory process [6–9].

*T. gondii* can infect all nucleated cells of birds and mammals, including humans, and consequently can cause cell lysis. Toxoplasmosis is of great health importance worldwide with a high prevalence that may cause congenital abnormalities such as hydrocephaly, abortion, jaundice, and chorioretinitis and severe disorders in immunocompromised subjects [10].

Moreover, based on results of many studies, latent toxoplasmosis seems to play an important role in the occurrence of a spectrum of neurological disorders, such as personality disorder, Parkinson disease, Alzheimer disease, and cryptic epilepsy [11]. The periodic rupture of tissue cysts is considered to be a source for release of specific cytokines and antibodies due to *Toxoplasma* infection [12]. Therefore, this study was aimed at determining the association between *T. gondii* infection and type I diabetes.

## 2. Materials and Methods

Ninety-one patients with type I diabetes, confirmed by physicians in endocrine clinics affiliated to the Shiraz University of Medical Sciences in Shiraz, Southern Iran, were enrolled.

Moreover, 91 sera samples were taken from healthy individuals as the control. The age of diabetic and control subjects was identical and less than 30 years. The samples were transferred to the serology laboratory at the Department of Parasitology and Mycology in Shiraz University of Medical Sciences. The sera were all stored at -70 till use for further studies. A questionnaire was prepared and completed for each subject including information on age, sex, and family history of diabetes.

An ELISA test was performed to determine chronic *Toxoplasma* infection using a *T. gondii* IgG ELISA kit (Pishtaz Teb Company, Tehran, Iran) according to the manufacturer instructions. Briefly, the test was based on an indirect ELISA technique wherein serum samples were allowed to react with coated *T. gondii* antigens. After incubation, the anti-human IgG that was conjugated with HRP was transferred into a well [13].

If anti-*T. gondii* IgG was present in samples, they reacted with the HRP-conjugated anti-human IgG. After incubation and appropriate washing, a solution of chromogen substrate was added and incubated for 15 minutes, resulting into development of a blue color. The color development was then stopped while the color changed into yellow. The solution was then evaluated by a spectrophotometer at 450 nm. The concentration of *T. gondii* IgG was directly proportional to the color intensity of the test samples.

Also, an ELISA test was performed to determine acute *Toxoplasma* infection using a *T. gondii* IgM ELISA kit (Pishtaz Teb Company, Tehran, Iran) according to the manufacturer instructions. Briefly, the test was based on an antibody capture technique in which microplate wells were coated with anti-human IgM antibodies. Diluted serum samples were allowed to react with coated antibodies. After incubation, all serum IgM molecules attached to the coated antibodies and unbound IgM antibodies were then removed by washing. To determine the specific anti-*T. gondii* IgM level, the *T. gondii* antigen that was conjugated with HRP was transferred into wells which were bound to their specific IgM antibody.

After incubation and appropriate washing, a solution of chromogen substrate was added and incubated for 15 minutes, resulting in the development of a blue color. The color development was then stopped while the color changed to yellow. The solution was then evaluated by spectrophotometry at 450 nm. The concentration of *T. gondii* IgM was directly proportional to the color intensity of the test sample.

## 3. Statistical Analysis

SPSS software (Version 16, Chicago, IL, USA) was used for analyzing of the data using chi-square and Student's *t*-tests. If the *P* value was less than 0.05, the difference between groups was considered significant.

## 4. Results

Out of 91 diabetic patients, 54 (59.3%) were female and 37 (40.7%) were male (Table 1). The highest frequency of diabetes was noticed among the 6 to 10- and 11 to 15-year-old patients (*P* value = 0.17) (Table 2). According to Table 3, a total of 33 cases (18.1%) had an IgG antibody to *Toxoplasma gondii*. This prevalence for the diabetic group was 28.6% and for the control group was 7.7% (*P* = 0.001).

There was no correlation between diabetes and acute Toxoplasma infection because all cases of both groups had no IgM antibody to the parasite. Also, no association was visible between acute toxoplasmosis and HbA1c as seven patients of acute toxoplasmosis (IgM positive) were evaluated in HbA1c. The HbA1c level was normal among all cases. As Table 4 shows, a positive family history of type I diabetes was observed among 31 cases (34.1%) of diabetic patients, and for the control group, it was 3 cases (3.3%). The difference between the two groups was statistically significant (*P* = 0.01).

IgG positivity was seen in 13 case (41.9%) of patients with positive family history of type I diabetes and 13 cases (21.7%) of subjects with no positive family history of type I diabetes (*P* = 0.043), but in the control group, the difference was not statistically significant (*P* = 1) (Table 5). IgG positivity was demonstrated among 26 cases of type I diabetes patients wherein 16 cases (29.6%) were female and 10 cases (27%) were male. No statistically significant difference in the infection and diabetes was found between both genders (*P* > 0.05) (Table 6). The peak prevalence of toxoplasmosis among diabetic patients was noticed in the 16-20 age group (62.5%), and the difference was statistically significant (*P* = 0.007) (Table 7).

## 5. Discussion

In this study, the correlation among *T. gondii* infection and age, sex, type I diabetes, and family history of the diabetes was evaluated using the ELISA test. Similar to our study, there were studies in Canada, Brazil, and Taiwan that showed that women had a significantly higher positivity rate than men [14–16]. However, numerous studies in Laos, Algeria, Uganda, and Saskatchewan of Canada denoted to an equal prevalence of type I diabetes in both sexes [17–20].

In the present study, the peak prevalence of type I diabetes was seen in 6-10 and 11-15 year age groups. Another study in Southwestern Saudi Arabia demonstrated that the prevalence of type I diabetes was 27.6% in children 5 years or less, while this prevalence was 72.4% in the group aged 8–12 years old [21]. The studies of Denmark and Sweden indicate a higher prevalence of type I diabetes in the 11-13-year-old age group among boys and the 9-11-year-old age group among girls [22, 23].

Our study indicated that a positive family history of type I diabetes was observed among 31 cases (34.1%) of diabetic patients, and for the control group, it was 3 cases (3.3%). One study in Iran displayed that this rate was 33.6% [24]. The result of a retrospective study in America showed that 25% of diabetic patients had a positive family history of diabetes. Also, factors such as depressed affect or negative affect could not influence diabetes incidence of the patient with positive parental history of diabetes [25].

In this study, anti-Toxoplasma IgG positivity was 18.1% in all cases. This prevalence in diabetic patients (28.6%) was more than that in the control group (7.7%). Similar to our study, case-control study results by Nassief Beshay et al. showed a higher seropositivity of anti-Toxoplasma IgG in diabetes mellitus I in comparison with the control group [26].

Furthermore, the results of two studies in China and Egypt showed that the diabetic patients have a higher seropositivity for anti-Toxoplasma IgG antibodies in comparison with the control [27, 28].

However, Khalili et al. did not detect any statistical differences among diabetic patients and the control in terms of toxoplasmosis [29].

Also, based on a systematic review and meta-analysis, no statistically significant correlation was seen between Toxoplasma infection and type I diabetes mellitus [30].

Of course, a few studies have been investigated to find the correlation between Toxoplasma and TIDM, and further research is warranted for this hypothesis.

The present study reports the anti-Toxoplasma IgG positivity of 7.7% in the control group, but other studies in Iran showed that IgG positivity in the 0-20-year-old age group was 28.7% and in the 1-30-year-old age group was 16.1% [31, 32]. In our study, anti-Toxoplasma IgM positivity like another study in Iran among pregnant women was relatively low (1.4%) [33].

Our findings showed that the prevalence among diabetic patients with IgG positivity increased with age until age of 20 years, and in the control group, the prevalence also showed an increasing trend in relation to age. Yang et al. reported that the prevalence among subjects with IgG positivity also rose with age [34]. In the study in Meshkinshahr, Iran denoted to an increasing prevalence among subjects with IgG positivity until 10 years old while declining at age of 30 years old [35].

In our study, no significant difference was observed between *T. gondii* infection and sex whereas the study in the Chaharmahal and Bakhtiari province in Iran showed that this rate was higher in women [36]. In Ireland, IgG positivity for Toxoplasma among students at the age of 4-18 years old was 12.8%, and the difference was not significant regarding sex [37]. In our study, the positive titer of IgG was observed in 41.9% of diabetic patients who had a positive family history of type 1 diabetes showing that *T. gondii* may be responsible in inducing type I diabetes in subjects with positive family history who were genetically predisposed to diabetes.

Our results showed that Toxoplasma infection may have a possible role in inducing type I diabetes in subjects who have a positive family history of the disease and are genetically predisposed to diabetes.

Based on our results, it seems that *T. gondii*, the same as a virus, may have a trigger role in the induction of type I diabetes in subjects who have a positive family history of the disease [3].

Remarkably, an association was reported between the appearance of the autoantibodies and enterovirus infection both among diabetic children and offspring with HLA-susceptibility to diabetes [38]. Also, many studies on rota-, entero-, cytomegalo-, and measles viruses have shown a correlation between viral infection and diabetes [39–41].

Since two decades ago, it has been thought that type I diabetes is a genetic autoimmune disorder, but the results of some researchers showed a low concordance of the disease among monozygotic twins supporting the role of nongenetic factors in the occurrence of type I diabetes [42]. The disease is an autoimmune disorder that is caused by infiltration of inflammatory cells in islets of Langerhans, consequently destructing the insulin-producing *β*-cells. The cell infiltration in the pancreatic Langerhans islet cells may be induced primarily by CD8+ T-cells and B-cells, followed by macrophages and dendritic cells [3].

The infection can induce a strong inflammation that is caused by activation of natural killer (NK) cells within the islets and initiate the induction of an autoimmune response [43]. It is not yet clear whether these pathogens can promote a direct cytolysis in *β*-cells or if their antigens can introduce autoantibodies against the antigen surface on *β*-cells [44].

## 6. Conclusion

Based on the current study, a correlation was noticed between Toxoplasma IgG seropositive and type I diabetic individuals. The parasite capacity in the stimulation of the disorder must be assessed by researches on animal models and must be severely controlled for the population at risk.

## Figures and Tables

**Table 1:** Number of subjects enrolled in the study.

Group	Sex	
	Female no. (%)	Male no. (%)
Diabetic	54 (59.3)	37 (40.7)
Control	58 (63.7)	33 (36.3)
Total	112 (61.5)	70 (38.5)

**Table 2:** Age range among the groups.

Group	Age range (years old)					
	0-5	6-10	11-15	16-20	21-25	26-30
Diabetic no. (%)	11 (12.1)	27 (29.7)	27 (29.7)	16 (17.6)	6 (6.6)	4 (4.4)
Control no. (%)	5 (5.5)	25 (27.5)	21 (23.1)	19 (20.9)	16 (17.6)	5 (5.5)
Total	16 (8.8)	52 (28.6)	48 (26.4)	35 (19.2)	22 (12.1)	9 (4.9)

**Table 3:** Anti-*Toxoplasma* IgG infection distribution among groups.

Group	Anti-*Toxoplasma* IgG	
	Negative no. (%)	Positive no. (%)
Diabetic	65 (71.4)	26 (28.6)
Control	84 (92.3)	7 (7.7)
Total	149 (81.9)	33 (18.1)

**Table 4:** Family history distribution for type 1 diabetes among both groups.

Group	Family history	
	Negative no. (%)	Positive no. (%)
Diabetic	60 (65.9)	31 (34.1)
Control	88 (96.7)	3 (3.3)
Total	148 (81.3)	34 (18.7)

**Table 5:** Anti-*Toxoplasma* IgG and family history distribution in both groups.

IgG	Group with positive family history of diabetes			
	Diabetic no. (%)		Control no. (%)	
	Positive	Negative	Positive	Negative
Positive	13 (41.9)	13 (21.7)	0 (0)	7 (8)
Negative	18 (51.8)	47 (78.3)	3 (100)	81 (92)
Total	31 (100)	60 (100%)	3 (100)	88 (100)

**Table 6:** Anti-*Toxoplasma* IgG and sex frequency among both groups.

IgG status	Group no. (%)			
	Diabetic no. (%)		Control no. (%)	
	Female	Male	Female	Male
Positive	16 (29.6)	10 (27)	2 (3.4)	5 (15.2)
Negative	38 (70.4)	27 (73)	56 (96.6)	28 (84.8)
Total	54 (100)	37 (100)	58 (100)	33 (100)

**Table 7:** Anti-*Toxoplasma* IgG and age group distribution in both groups.

Age (years)	Group			
	Diabetic		Control	
	Negative IgG no. (%)	Positive IgG no. (%)	Negative IgG no. (%)	Positive IgG no. (%)
0-5	11 (100)	0 (0)	5 (100)	0 (0)
6-10	21 (77.8)	6 (22.2)	25 (100)	0 (0)
11-15	19 (70.4)	8 (29.6)	18 (85.7)	3 (14.3)
16-20	6 (37.5)	10 (62.5)	19 (100)	0 (0)
21-25	4 (66.7)	2 (33.3)	13 (81.25)	3 (18.8)
26-30	4 (100)	0 (0)	4 (80)	1 (20)
Total	65 (71.4)	26 (28.6)	84 (92.3)	7 (7.7)

## Data Availability

The data used to support the findings of this study are available from the corresponding author upon request.
